# Initial results of a new very rapid rest/regadenoson stress myocardial perfusion protocol in patients with atrial fibrillation

**DOI:** 10.1186/1532-429X-16-S1-M6

**Published:** 2014-01-16

**Authors:** Lowell Chang, Promporn Suksaranjit, Gangadhar Malasana, Allen Rassa, Ganesh Adluru, Krishna Velagapudi, Devavrat Likhite, Alexis Harrison, Brent D Wilson, Christopher J McGann, Nassir F Marrouche, Edward V DiBella

**Affiliations:** 1 Cardiology, The University of Utah School of Medicine, Salt Lake City, Utah, USA; 2 Utah Center for Advanced Imaging Research, The University of Utah School of Medicine, Salt Lake City, Utah, USA

## Background

Cardiovascular magnetic resonance (CMR) myocardial perfusion is a well established method for detection of significant obstructive coronary artery disease (CAD). In patients with arrhythmias, standard methods using ECG-gating can result in poor image quality. Additionally, with typical stress/rest protocols, a true rest state may not be achieved after administration of regadenoson. However, rest-first may present issues with peri-infarct ischemia and so here we give little time for late enhancement by keeping rest and stress perfusion scans close in time. Given these issues, the two-fold aim of this study is to evaluate the accuracy of a rapid rest-first protocol using an ungated myocardial image pulse sequence.

## Methods

**Figure 1:**
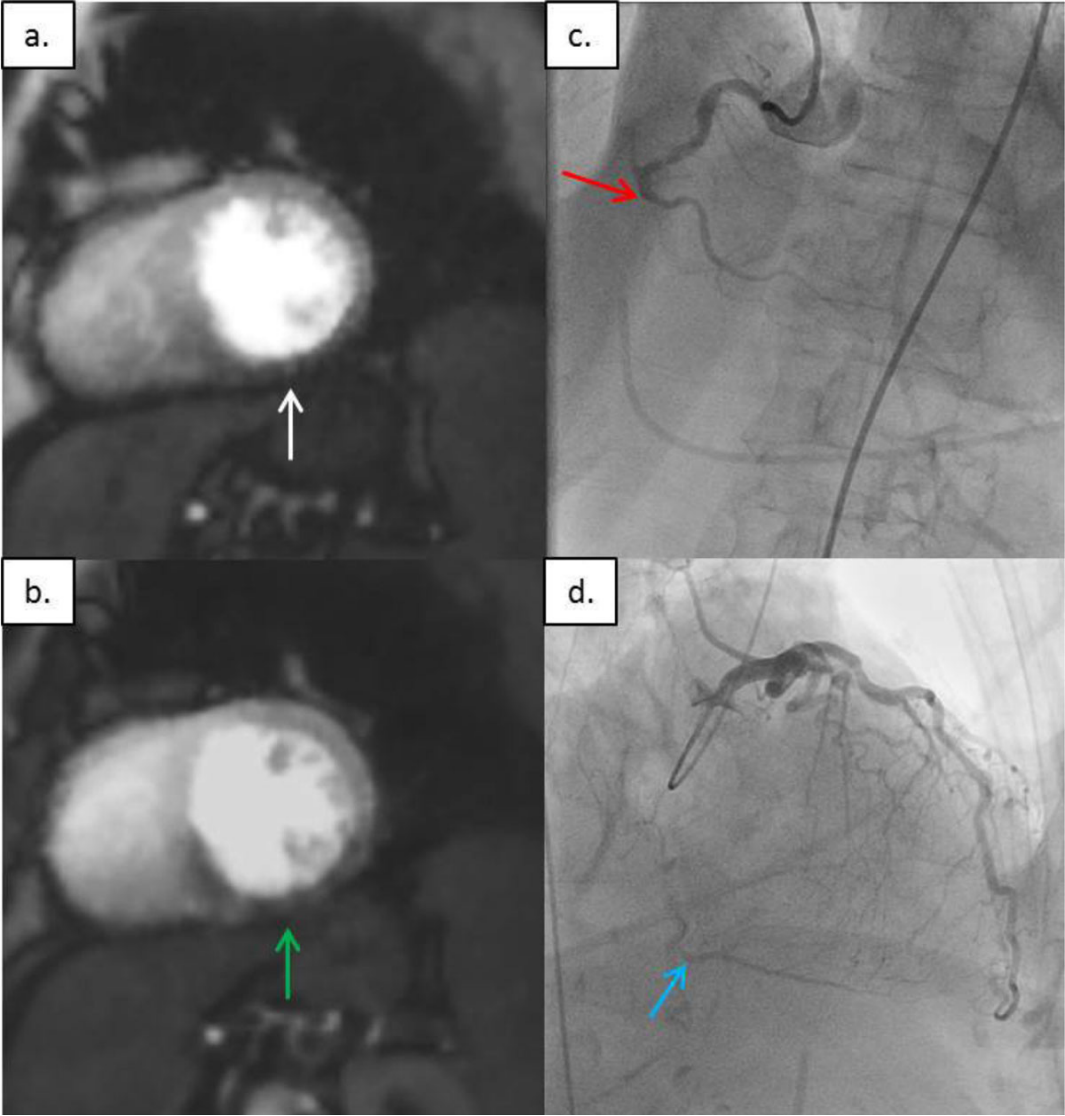
**Rest and stress perfusion images showing inferior wall defect with corresponding coronary x-ray angiography**. a. Rest perfusion image demonstrating mid inferior wall defect (white arrow). b. Stress perfusion image demonstrating mid inferior wall defect with septal wall extension (green arrow). c. Coronary X-ray angiography demonstrating chronic total occlusion of the mid right coronary artery (red arrow). d. Coronary X-ray angiography demonstrating distal right coronary artery filling via collaterals (blue arrow).

This prospective, single-blinded study included seven atrial fibrillation patients who underwent ungated rest/stress perfusion imaging and coronary x-ray angiography. Images were acquired using an ungated radial myocardial perfusion sequence (TR/TE = 2.2/1.2 msec, 3T, 20 rays/slice, 5 slices after each saturation pulse, ~2 × 2 × 8 mm), as described in a previous adenosine stress-first study. Rest/stress protocol was performed in the following fashion: rest image acquisition (0.05 mmol/kg gadoteridol, 1.5 minutes), administration of regadenoson 0.4 mg intravenously (0.4 mg/5 mL) to induce hyperemia, 70 second wait, then stress image acquisition (0.075 mmol/kg gadoteridol, 1.5 minutes). CMR images were interpreted (0 = normal, 1 = equivocal, but probably normal, 2 = probable ischemia, 3 = definitely abnormal) and evaluated for quality (1 to 5, lowest to highest quality) by two blinded readers. Perfusion results were condensed to normal (0-1) or disease (2-3). 14 readings for 7 patients were derived from separate reader results. CMR perfusion diagnostic accuracy for the detection of ischemic heart disease was determined by comparison to x-ray angiography with significant lesions defined as ≥70% stenosis or FFR≤0.8 (Figure 1).

## Results

Sensitivity and specificity of this CMR perfusion in the detection of significant coronary lesions were 1 and 0.88, respectively. Average quality of the readings was 3.8 ± 0.8 for both rest and stress perfusion images. Average scan-time for rest/stress perfusion imaging acquisition including time of pharmaceutical injection was 6.5 ± 4.0 minutes.

## Conclusions

Initial results for this ongoing rest/regadenoson stress protocol using an ungated myocardial perfusion sequence yielded high sensitivity and specificity for the detection of significant CAD with good image quality. This combination of a novel protocol and an ungated radial sequence addresses the concerns of lingering hyperemia with regadenoson along with problematic gating in arrhythmias.

## Funding

Astellas Pharma Inc.

